# ”HOW OLD IS OLD?” ADDRESSING AGEISM AND UNCONSCIOUS BIAS AMONG MEDICAL STUDENTS DURING GERIATRICS CLERKSHIP

**DOI:** 10.1093/geroni/igac059.1268

**Published:** 2022-12-20

**Authors:** Ravishankar Ramaswamy, Stephanie Chow, Noelle Marie Javier, Rosanne Leipzig, Gregory Hinrichsen, Amy Kelley

**Affiliations:** Icahn School of Medicine at Mount Sinai, New York, New York, United States; Icahn School of Medicine at Mount Sinai, New York, New York, United States; Icahn School of Medicine at Mount Sinai, New York, New York, United States; Icahn School of Medicine at Mount Sinai, New York, New York, United States; Icahn School of Medicine at Mount Sinai, New York, New York, United States; Icahn School of Medicine at Mount Sinai, New York, New York, United States

## Abstract

Ageism (stereotyping, prejudice and discrimination based on age) has deleterious consequences on older adults’ health. Medical students have variable attitudes and biases toward older people. We hypothesized that an embedded ageism curriculum within the Ambulatory Care-Geriatrics clerkship would increase ageism awareness and commitment to reduce ageism in the clinical environment for third year medical students. The 2021 curriculum included assigned pre-reading, videos, short didactics, expert-facilitated small group discussion of clinical vignettes, reflective journaling, and posting of personal commitments on a virtual messaging board. We surveyed students at the start and end of the clerkship to evaluate baseline awareness, change in UCLA Geriatrics Attitudes Scale, and satisfaction with curricular components. Of the 95 students who thusfar participated in the curriculum, we received 92 pre- and 48 post-curriculum survey responses. Pre-curriculum students reported the median age for “old” was 65 years (range 35-90) and 42% of students expressed preference for younger patients (33% neutral). Proportion of students with self-assessed ability to identify ageist remarks/actions increased from 52%(pre) to 92%(post), and ability to minimize own ageist biases increased from 23%(pre) to 83%(post). 86% of students found the curriculum useful; discussion with experts and viewing an ageism TED Talk were the most favorably scored components. Integrating an ageism curriculum with pre-work, didactic, guided discussion and reflection components in the Geriatrics clerkship increased medical student awareness and confidence in addressing ageism-related behaviors. This curriculum complements students’ clinical interactions with older adults and has the potential to reduce the future impact of ageism in medicine.

